# Co(NCS)_2_(abpt)_2_ and Ni(NCS)_2_(abpt)_2_ [abpt is 4-amino-3,5-bis­(pyridin-2-yl)-1,2,4-triazole]: structural characterization of polymorphs A and B

**DOI:** 10.1107/S2053229621010251

**Published:** 2021-11-16

**Authors:** Helen E. Mason, Judith A. K. Howard, Hazel A. Sparkes

**Affiliations:** a Department of Chemistry, Durham University, South Road, Durham DH1 3LE, United Kingdom; b School of Chemistry, University of Bristol, Cantock’s Close, Bristol BS8 1TS, United Kingdom

**Keywords:** polymorphism, abpt, crystal structure, 4-amino-3,5-bis­(pyridin-2-yl)-1,2,4-triazole

## Abstract

The synthesis and structures of bis­[4-amino-3,5-bis­(pyridin-2-yl)-1,2,4-triazole-κ^2^ *N* ^2^,*N* ^3^]bis­(thio­cyanato-κ*N*)cobalt(II), [Co(NCS)_2_(C_12_H_10_N_6_)_2_] or Co(NCS)_2_(abpt)_2_, and bis­[4-amino-3,5-bis­(pyridin-2-yl)-1,2,4-triazole-κ^2^ *N* ^2^,*N* ^3^]bis­(thio­cyan­ato-κ*N*)nickel(II), [Ni(NCS)_2_(C_12_H_10_N_6_)_2_] or Ni(NCS)_2_(abpt)_2_, are reported. In both cases, two polymorphs, **A** and **B**, were identified and structurally characterized. For both polymorphs, the structures obtained with the different metals, *i.e.* Co^II^ or Ni^II^, were found to be isostructural. All of the structures contained an intra­molecular N—H⋯N hydrogen bond, C—H⋯N inter­actions and π–π stacking inter­actions. No structural evidence was observed for a thermal spin crossover for either of the Co(NCS)_2_(abpt)_2_ polymorphs between 300 (2) and 120 (2) K.

## Abstract 2

The synthesis and structures of two polymorphs, **A** and **B**, of Co(NCS)_2_(abpt)_2_ and Ni(NCS)_2_(abpt)_2_ are reported. The polymorph structures of **A** with Co^II^ or Ni^II^ were found to be isostructural, as were the corresponding pair of polymorph **B** structures with the different metals.

## Introduction

The bidentate ligand 4-amino-3,5-bis­(pyridine-2-yl)-1,2,4-tri­azole (abpt) has been found to form mononuclear com­plexes, as well as single- or double-bridged dinuclear com­plexes, with a variety of metals (for examples, see Dupouy *et al.*, 2008 ▸; White *et al.*, 2009 ▸; Li *et al.*, 2011 ▸). Amongst these, a number of Fe^II^ com­plexes have been synthesized and studied because of their inter­esting polymorphism and spin-crossover behaviour. Perhaps the most studied is the Fe(NCS)_2_(abpt)_2_ com­plex, of which there are four known polymorphs, denoted **A**–**D**, all of which display different magnetic behaviour. Three of the polymorphs, *i.e.* **A** (Moliner *et al.*, 1999 ▸; Sheu *et al.*, 2009 ▸; Mason *et al.*, 2016 ▸), **C** (Sheu *et al.*, 2009 ▸; Shih *et al.*, 2010 ▸) and **D** (Sheu *et al.*, 2009 ▸, 2012 ▸; Mason *et al.*, 2021 ▸), undergo at least a partial thermal spin crossover under ambient pressure, while polymorph **B** (Gaspar *et al.*, 2003 ▸) only undergoes a thermal spin crossover at pressures above 4.4 kbar (1 bar = 10^5^ Pa). All of the three polymorphs which display at least a partial thermal spin crossover also show light-induced excited-spin-state trapping (LIESST) at low temperature. While three of the polymorphs (**A**, **B** and **D**) are known to undergo a pressure-induced spin crossover at room temperature (Mason *et al.*, 2016 ▸, 2021 ▸), polymorph **C** has not been studied under pressure at room temperature. To date, Co(NCS)_2_(abpt)_2_ is the only other *M*(NCS)_2_(abpt)_2_ com­plex containing a transition metal for which any structures have been reported. Like the Fe analogue, this has also been found to display polymorphism, with two different polymorphs of Co(NCS)_2_(abpt)_2_ reported at room temperature. These will be referred to as Co(NCS)_2_(abpt)_2_ polymorphs **B** (Peng *et al.*, 2006 ▸) and **D** (Chen & Peng, 2007 ▸) throughout, as they are isostructural with Fe(NCS)_2_(abpt)_2_ polymorphs **B** and **D**. The structures of two polymorphs, **A** and **B**, of both Co(NCS)_2_(abpt)_2_ and Ni(NCS)_2_(abpt)_2_ are reported herein (see Scheme 1). 
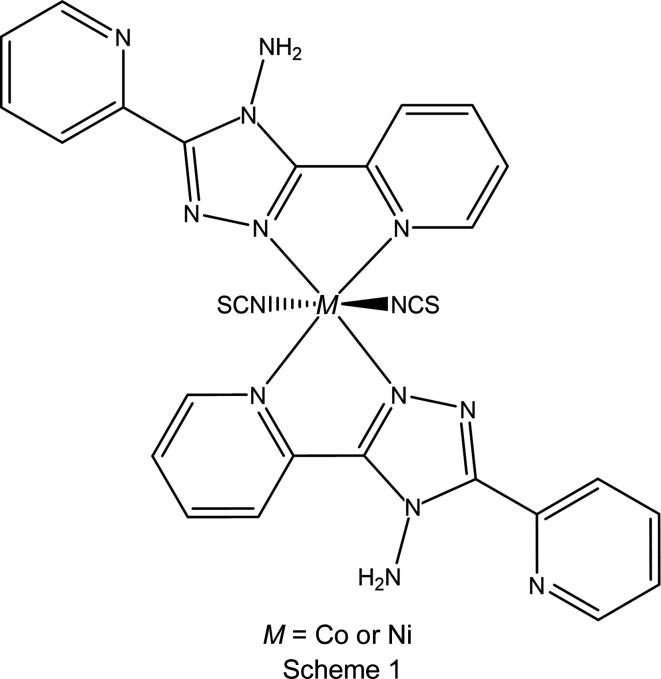


## Experimental

### Synthesis

The synthesis of *M*(NCS)_2_(abpt)_2_, where *M* is Co or Ni, was carried out using a slow-diffusion method with methanol–water solutions as reported previously (Sheu *et al.*, 2009 ▸).

All chemicals were obtained from Sigma–Aldrich and used as supplied. CoSO_4_·7H_2_O (1 mmol, 0.281 g) or NiSO_4_·6H_2_O (1 mmol, 0.263 g) and KNCS (2 mmol, 0.194 g) were stirred in methanol (10 ml) for 15 min. A pale-yellow insoluble K_2_SO_4_ precipitate was removed by filtration and deionized water (10 ml) was added to the remaining clear solution. Abpt (2 mmol, 0.477 g) was dissolved in methanol (20 ml) and placed in a narrow (<5 cm) Schlenk tube. The *M* ^2+^/NC*X* ^−^ solution was very carefully pipetted at the bottom of the Schlenk tube to form a lower more dense layer below the abpt solution. Immediately, a coloured band formed at the inter­face between the two layers containing the target com­plex. The Schlenk tube was left undisturbed and single crystals suitable for X-ray diffraction studies had formed within one week to one month later.

### Refinement

Details of the crystallographic data collections are given in Table 1 ▸. All H atoms, apart from the N—H hydrogens, were positioned geometrically and refined using a riding model. The N—H hydrogens were located in a difference Fourier map (FDM) wherever feasible.

## Results and discussion

**Figure 1:**
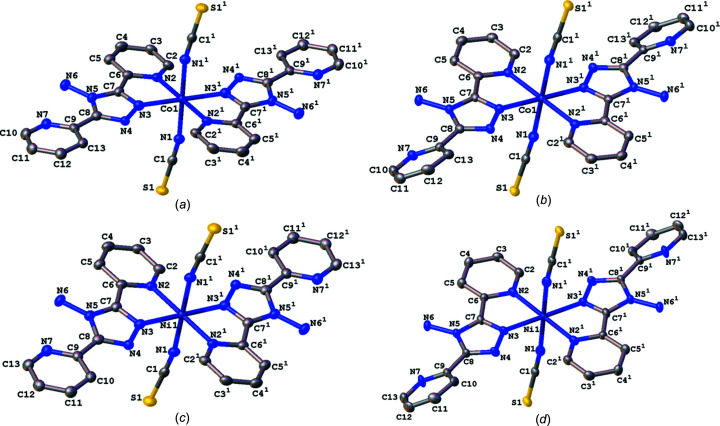
Illustration of the structures of Co(NCS)_2_(abpt)_2_ polymorphs (*a*) **A** and (*b*) **B**, and Ni(NCS)_2_(abpt)_2_ polymorphs (*c*) **A** and (*d*) **B**, with the atomic numbering schemes depicted. H atoms have been omitted for clarity. [Symmetry code: (i) −*x* + 1, −*y* + 1, −*z* + 1.]

The structure of Co(NCS)_2_(abpt)_2_ polymorph **B** has already been reported at room temperature and is consistent with that reported here (Peng *et al.*, 2006 ▸). The main structural features of all four structures are very similar: they all crystallized in the monoclinic space group *P*2_1_/*n* with half a mol­ecule in the asymmetric unit (*Z*′ = 0.5) (Fig. 1 ▸). Each of the four com­plexes consists of an approximately octa­hedrally coordinated metal centre (Co^II^ or Ni^II^) coordinated to six N atoms, one from each of the NCS^−^ ligands and two from each abpt ligand (one pyridyl and one triazole N atom). Each of the structures contains an intra­molecular N—H⋯N hydrogen bond between the NH_2_ group on the triazole ring and the N atom of the uncoordinated pyridyl ring, as well as two intra­molecular C—H⋯N inter­actions, one between a pyridyl C—H group and the N atom of the NH_2_ group attached to the triazole ring, and a second between a pyridyl C—H group and the uncoordinated N atom on the triazole group (Table 2 ▸).

**Figure 2:**
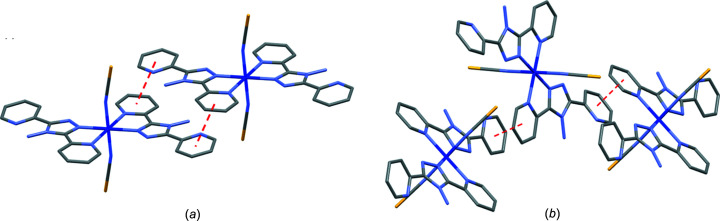
Illustration of the π–π stacking inter­actions as red dashed lines for one abpt ligand in (*a*) polymorph **A** (*b*) polymorph **B**.

The pair of **A** polymorphs of the Co^II^ or Ni^II^ structures are isostructural with each other, and are also isostructural with the previously reported Fe(NCS)_2_(abpt)_2_ polymorph **A** structure (Moliner *et al.*, 1999 ▸; Sheu *et al.*, 2009 ▸; Mason *et al.*, 2016 ▸). In addition to the previously mentioned N—H⋯N hydrogen bonding and C—H⋯N inter­actions, the structures contain inter­molecular π–π stacking between pairs of mol­ecules and involving the two pyridyl rings at each end of the abpt ligand inter­acting with the two pyridyl rings on an adjacent abpt ligand, creating a one-dimensional chain through the structure (Table 3 ▸ and Fig. 2 ▸).

As seen for the pair of polymorph **A** structures, the two polymorph **B** structures were also isostructural with each other and with the previously reported Fe(NCS)_2_(abpt)_2_ polymorph **B** structure (Gaspar *et al.*, 2003 ▸; Mason *et al.*, 2021 ▸). The structures of polymorph **B** also display π–π inter­actions, but in this case each of the pyridyl rings on the abpt ligand is involved in a π–π inter­action to a pyridyl ring on a different abpt ligand, creating a three-dimensional network of inter­actions in the structure (Table 3 ▸ and Fig. 2 ▸). Along with the difference in the form of the π–π inter­actions between the polymorph **A** and polymorph **B** structures, the other main difference is the twist between the two rings on the abpt ligands. In the case of **A**, the twist between the rings is ∼9°, while for **B**, the twist between the rings is ∼35° (Table 4 ▸). This is likely to be the reason for the significantly different π–π stacking, as the larger twist in **B** would prevent both rings on one abpt ligand being correctly orientated to inter­act with both rings on a single abpt ligand on an adjacent mol­ecule.

**Figure 3:**
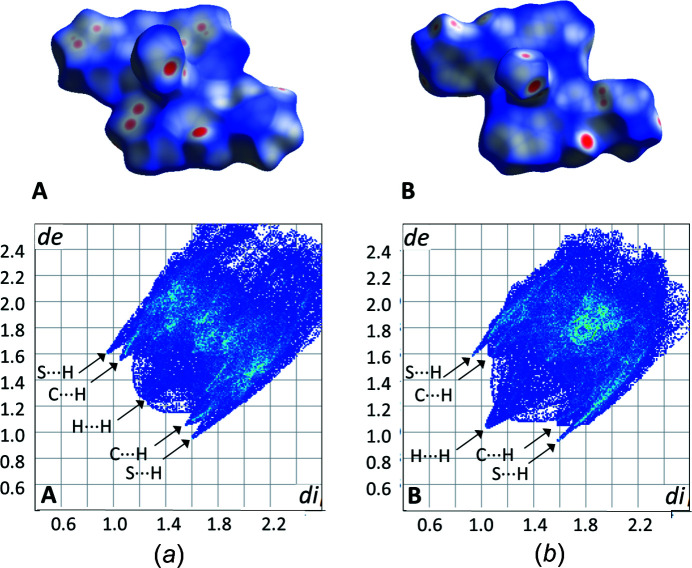
The Hirshfeld surface plot and fingerprint plot for (*a*) polymorph **A** and (*b*) polymorph **B** for Co(NCS)_2_(abpt)_2_. The Ni plots for the same respective polymorphs are essentially identical.

The Hirshfeld fingerprint plots (Turner *et al.*, 2017 ▸) for the two polymorphs highlight the differences between the two structures (Fig. 3 ▸). The plots are only shown for the Co polymorphs **A** and **B**, as the plots for the Ni polymorphs **A** and **B** were essentially identical to those of the respective Co polymorphs. The shapes of the two plots are clearly slightly different, although given that the structures are polymorphs, it is unsurprising that they show the same main short con­tacts. For both polymorphs, the S⋯H con­tacts are quite pro­noun­ced, with a similar shape and position. However, in the case of **A**, the C⋯H con­tacts are more pronounced than is seen for **B**, while the H⋯H con­tacts for **A** are less pro­nounced than observed for **B**. Examining the Hirshfeld surfaces for both com­pounds, the greater number of red spots on the surface of **A** than for **B** indicates that **A** has more short con­tacts.

Given that Fe(NCS)_2_(abpt)_2_ polymorph **A** was shown to have a spin transition upon cooling (Moliner *et al.*, 1999 ▸; Sheu *et al.*, 2009 ▸; Mason *et al.*, 2016 ▸), the data for Co^II^ *d* ^7^ polymorphs **A** and **B** were also measured at 300 (2) K (Table S1 in the supporting information). Examining the Co—N bond lengths showed them to be essentially identical to the 120 (2) K structure and indicate that no spin transition had occurred over this temperature range (Table 5 ▸). In the case of Ni^II^, the com­plex is *d* ^8^ so no spin transition would be possible.

## Conclusions

The synthesis and structures of Co(NCS)_2_(abpt)_2_ and Ni(NCS)_2_(abpt)_2_ are reported. Two polymorphs were identified for each of the com­plexes, **A** and **B**, and the pairs of polymorphs with the different metal centres were found to be isostructural. All of the structures contained intra­molecular N—H⋯N hydrogen bonding, intra­molecular C—H⋯N inter­actions and π–π stacking. There are identifiable differences between the two polymorph structures. Firstly, the twist angle between the two six-membered rings on one abpt ligand was ∼9° for polymorph **A** and ∼35° for polymorph **B**. Secondly, the nature of the π–π stacking inter­actions was significantly different, presumably due to the differing twist angles of the rings. In the case of **A**, both rings on one abpt ligand form π–π stacking inter­actions with both rings on an abpt ligand on an adjacent mol­ecule, while for **B**, each of the rings on the abpt ligand forms π–π stacking inter­actions with a ring on different abpt ligands in adjacent mol­ecules. Variable-temperature studies on *d* ^7^ Co(NCS)_2_(abpt)_2_ did not show any evidence of a thermally-induced spin crossover for either of the polymorphs between 300 (2) and 120 (2) K.

## Supplementary Material

Crystal structure: contains datablock(s) Co_A_120K, Co_B_120K, Ni_A_120K, Ni_B_120K, Co_A_300K, Co_B_300K, global. DOI: 10.1107/S2053229621010251/oc3012sup1.cif

Structure factors: contains datablock(s) Co_A_120K. DOI: 10.1107/S2053229621010251/oc3012Co_A_120Ksup2.hkl

Structure factors: contains datablock(s) Co_B_120K. DOI: 10.1107/S2053229621010251/oc3012Co_B_120Ksup3.hkl

Structure factors: contains datablock(s) Ni_A_120K. DOI: 10.1107/S2053229621010251/oc3012Ni_A_120Ksup4.hkl

Structure factors: contains datablock(s) Ni_B_120K. DOI: 10.1107/S2053229621010251/oc3012Ni_B_120Ksup5.hkl

Structure factors: contains datablock(s) Co_A_300K. DOI: 10.1107/S2053229621010251/oc3012Co_A_300Ksup6.hkl

Structure factors: contains datablock(s) Co_B_300K. DOI: 10.1107/S2053229621010251/oc3012Co_B_300Ksup7.hkl

Additional crystallographic information. DOI: 10.1107/S2053229621010251/oc3012sup8.pdf

CCDC references: 2113662, 2113661, 2113660, 2113659, 2113658, 2113657

## Figures and Tables

**Table 1:** Experimental details For all structures: monoclinic, *P*2_1_/*n*, *Z* = 2. Experiments were carried out at 120 K with Mo *K*α radiation. Absorption was corrected for by multi-scan methods (*SADABS*; Bruker, 1999–2013 ▸). Refinement was on 202 parameters. H atoms were treated by a mixture of independent and constrained refinement.

	Co(NCS)_2_(abpt)_2_, Polymorph **A**	Co(NCS)_2_(abpt)_2_, Polymorph **B**	Ni(NCS)_2_(abpt)_2_, Polymorph **A**	Ni(NCS)_2_(abpt)_2_, Polymorph **B**
Crystal data				
Chemical formula	[Co(NCS)_2_(C_12_H_10_N_6_)_2_]	[Co(NCS)_2_(C_12_H_10_N_6_)_2_]	[Ni(NCS)_2_(C_12_H_10_N_6_)_2_]	[Ni(NCS)_2_(C_12_H_10_N_6_)_2_]
*M*_r_	651.61	651.61	651.39	651.39
*a*, *b*, *c* (Å)	8.4792 (6), 10.1307 (7), 16.3774 (11)	11.4978 (5), 9.5235 (4), 12.7179 (5)	8.4041 (7), 10.0681 (9), 16.2360 (14)	11.5860 (14), 9.5489 (12), 12.8132 (16)
β (°)	93.485 (1)	100.771 (1)	93.060 (2)	100.806 (2)
*V* (Å^3^)	1404.22 (17)	1368.07 (10)	1371.8 (2)	1392.4 (3)
μ (mm^−1^)	0.81	0.83	0.91	0.89
Crystal size (mm)	0.24 × 0.16 × 0.11	0.48 × 0.22 × 0.1	0.2 × 0.12 × 0.08	0.2 × 0.13 × 0.04

Data collection				
Diffractometer	Bruker SMART CCD 1K area detector	Bruker SMART CCD 1K area detector	Bruker D8 VENTURE	Bruker SMART CCD 1K area detector
*T*_min_, *T*_max_	0.793, 0.919	0.755, 0.884	0.781, 0.936	0.746, 0.948
No. of measured, independent and observed [*I* > 2σ(*I*)] reflections	13341, 2884, 2383	13084, 2799, 2363	15450, 2819, 2161	12077, 2552, 1666
*R*_int_	0.044	0.037	0.046	0.116
(sin θ/λ)_max_ (Å^−1^)	0.625	0.625	0.625	0.602

Refinement				
*R*[*F* ^2^ > 2σ(*F* ^2^)], *wR*(*F* ^2^), *S*	0.039, 0.093, 1.06	0.028, 0.065, 1.03	0.037, 0.085, 1.02	0.058, 0.136, 1.06
No. of reflections	2884	2799	2819	2552
No. of restraints	1	0	0	0
Δρ_max_, Δρ_min_ (e Å^−3^)	0.58, −0.27	0.26, −0.39	0.48, −0.27	0.61, −0.66

Computer programs: *SMART*, *APEX2*, *SAINT* and *SAINT-Plus* (Bruker, 1999–2013 ▸), *SHELXS* (Sheldrick, 2008 ▸), *SHELXL2018* (Sheldrick, 2015 ▸) and *OLEX2* (Dolomanov *et al.*, 2009 ▸).

**Table 2:** Hydrogen-bond geometry (Å, °) for Co(NCS)_2_(abpt)_2_ and Ni(NCS)_2_(abpt)_2_ at 120 (2) K

Structure	Polymorph	*D*—H⋯*A*	*D*—H	H⋯*A*	*D*⋯*A*	*D*—H⋯*A*
Co(NCS)_2_(abpt)_2_	**A**	N6—H6*B*⋯N7	0.90 (3)	2.14 (3)	2.861 (3)	136 (3)
		C5—H5⋯N6	0.95	2.53	3.135 (4)	122
		C2—H2⋯N4^i^	0.95	2.67	3.467 (3)	142
	**B**	N6—H6*B*⋯N7	0.90 (2)	2.41 (2)	2.914 (2)	115.6 (16)
		C5—H5⋯N6	0.95	2.46	3.084 (2)	123
		C2—H2⋯N2^i^	0.95	2.66	3.482 (2)	145
Ni(NCS)_2_(abpt)_2_	**A**	N6—H6*B*⋯N7	0.88 (3)	2.14 (3)	2.848 (3)	137 (3)
		C5—H5⋯N6	0.95	2.52	3.124 (4)	122
		C2—H2⋯N4^ii^	0.95	2.55	3.347 (3)	141
	**B**	N6—H6*B*⋯N7	0.84 (6)	2.52 (5)	2.950 (6)	112 (4)
		C5—H5⋯N6	0.95	2.48	3.104 (7)	123
		C2—H2⋯N4^ii^	0.95	2.59	3.403 (7)	144

Symmetry codes: (i) −*x* + 1, −*y* + 1, −*z* + 1; (ii) −*x* + 1, −*y* + 1, −*z* + 1.

**Table 3:** π–π stacking inter­actions (Å) for Co(NCS)_2_(abpt)_2_ and Ni(NCS)_2_(abpt)_2_ at 120 (2) K

Structure	Polymorph	Plane 1	Plane 2	Centroid-to-centroid distance	Shift distance
Co(NCS)_2_(abpt)_2_	**A**	N2,C2,C3,C4,C5,C6	N7,C9,C10,C11,C12,C13^i^	3.63	1.31
		N7,C9,C10,C11,C12,C13	N2,C2,C3,C4,C5,C6^i^	3.63	1.31
	**B**	N2,C2,C3,C4,C5,C6	N7,C9,C10,C11,C12,C13^ii^	3.68	1.34
		N7,C9,C10,C11,C12,C13	N2,C2,C3,C4,C5,C6^iii^	3.68	1.34
Ni(NCS)_2_(abpt)_2_	**A**	N2,C2,C3,C4,C5,C6	N7,C9,C10,C11,C12,C13^i^	3.64	1.34
		N7,C9,C10,C11,C12,C13	N2,C2,C3,C4,C5,C6^i^	3.64	1.34
	**B**	N2,C2,C3,C4,C5,C6	N7,C9,C10,C11,C12,C13^ii^	3.72	1.41
		N7,C9,C10,C11,C12,C13	N2,C2,C3,C4,C5,C6^iii^	3.72	1.41

Symmetry codes: (i) −*x* + 1, −*y* + 2, −*z* + 1; (ii) *x* + {1\over 2}, −*y* + {3\over 2}, *z* + {1\over 2}; (iii) *x* − {1\over 2}, −*y* + {3\over 2}, *z* − {1\over 2}.

**Table 4:** Twist and fold angles between planes calculated through the six atoms of the two rings on the abpt ligand at 120 (2) K

Compound	Polymorph	Twist angle (°)	Fold angle (°)
Co(NCS)_2_(abpt)_2_	**A**	8.99 (8)	99.0 (8)
	**B**	35.25 (6)	142.50 (19)
Ni(NCS)_2_(abpt)_2_	**A**	9.39 (8)	96.7 (8)
	**B**	34.64 (17)	142.8 (6)

**Table 5:** Co—N distances for Co(NCS)_2_(abpt)_2_ at 120 (2) and 300 (2) K

Polymorph	*T* (K)	Co1—N1	Co1—N2	Co1—N3
**A**	120	2.116 (2)	2.166 (2)	2.088 (2)
	300	2.113 (5)	2.164 (4)	2.093 (5)
**B**	120	2.0987 (15)	2.1616 (15)	2.1138 (14)
	300	2.102 (2)	2.166 (2)	2.1161 (18)
