# Supplementary material for: Co(NCS)2(abpt)2 and Ni(NCS)2(abpt)2 [abpt is 4-amino-3,5-bis­(pyridin-2-yl)-1,2,4-triazole]: structural characterization of polymorphs A and B
Source: Acta Crystallogr C Struct Chem. 2021 Nov 16;77(Pt 12):777–81. doi: 10.1107/S2053229621010251 (PMC8645156; doi:10.1107/S2053229621010251)
Supplement: Supplementary file 8 [file c-77-00777-sup8.pdf]

Acta Cryst C STRUCTURAL CHEMISTRY

Volume 77 (2021)

Supporting information for article:

Co(NCS)2(abpt)2 and Ni(NCS)2(abpt)2 [abpt is 4-amino-3,5-bis-(pyridin-2-yl)-1,2,4-triazole]: structural characterization of polymorphs A and B

Helen E. Mason, Judith A. K. Howard and Hazel A. Sparkes

Table S1 Experimental details at 300(2) K

	$Co(abpt)_2(NCS)_2$ Polymorph A	$Co(abpt)_2(NCS)_2$ Polymorph B
Crystal data		
Chemical formula	$C_{26}H_{20}CoN_{14}S_2$	$C_{26}H_{20}CoN_{14}S_2$
M_r	651.61	651.61
Crystal system, space group	Monoclinic, $P2_1/n$	Monoclinic, $P2_1/n$
Temperature (K)	300	300
a, *b*, *c* (Å)	8.487 (5), 10.249 (6), 16.539 (10)	11.5855 (6), 9.5998 (5), 12.8411 (6)
β (°)	93.419 (13)	101.300 (1)
V ($Å^3$)	1435.9 (14)	1400.48 (12)
Z	2	2
Radiation type	Mo *K*α	Mo *K*α
μ (mm^{-1})	0.79	0.81
Crystal size (mm)	0.24 × 0.16 × 0.12	0.48 × 0.22 × 0.1
Data collection		
Diffractometer	Bruker *SMART* CCD 1K area detector	Bruker *SMART* CCD 1K area detector
Absorption correction	Multi-scan *SADABS2008*/1 (Bruker,2008) was used for absorption correction. wR2(int) was 0.1268 before and 0.0594 after correction. The Ratio of minimum to maximum transmission is 0.7415. The λ/2 correction factor is 0.0015.	Multi-scan *SADABS2008*/1 (Bruker,2008) was used for absorption correction. wR2(int) was 0.0699 before and 0.0469 after correction. The Ratio of minimum to maximum transmission is 0.9080. The λ/2 correction factor is 0.0015.
T_{min}, T_{max}	0.683, 0.921	0.805, 0.887
No. of measured, independent and	7130, 2605, 1378	7562, 2565, 2034

observed [$I > 2\sigma(I)$] reflections		
R_{int}	0.087	0.031
$(\sin \theta/\lambda)_{max}$ (Å^{-1})	0.602	0.602
Refinement		
$R[F^2 > 2\sigma(F^2)]$, $wR(F^2)$, S	0.059, 0.166, 1.02	0.034, 0.079, 1.03
No. of reflections	2605	2565
No. of parameters	202	202
No. of restraints	0	0
H-atom treatment	H atoms treated by a mixture of independent and constrained refinement	H atoms treated by a mixture of independent and constrained refinement
$\Delta\rho_{max}$, $\Delta\rho_{min}$ (e Å^{-3})	0.36, −0.47	0.22, −0.27

Computer programs: *SMART* v5.049 (Bruker, 1999), *SAINT* v6.45A (Bruker, 2003), *APEX2* (Bruker, 2005), *APEX2 SAINT*+ V8.32B (Bruker, 2013), *SHELXS* (Sheldrick, 2008), *SHELXS1997* (Sheldrick, 2008), *SHELXL* 2018/3 (Sheldrick, 2015), XL (Sheldrick, 2008), Olex2 1.3 (Dolomanov *et al.*, 2009).
